# W. J. H. Pinniger, M.D.

**Published:** 1917-12

**Authors:** 


### DR. W. J. H. PINNIGER.

The death of Dr. W. J. H. Pinniger, which occurred at his home in Clifton on August 4th last, cut short at the early age of 34 the career of one who, had he lived, was destined to attain a leading position in the Bristol medical world. The news of his death came as a great shock to the many who claimed his friendship, a shock which was only in a measure tempered by the fact that it was not unexpected.

Dr. Pinniger was a son of the late Thomas Large Pinniger, of Beckhampton, Wilts., and of Mrs. Pinniger, of Clifton. He was educated at Colchester House School, Clifton College, and University College, Bristol, spending some time later in medical work in London at King's and University College Hospitals.

As a student at the General Hospital he gained the Committee's Gold Medal for the best student of his year. He obtained the Conjoint qualification in July, 1905, and the M.B., B.S. Lond. the same year, proceeding to his M.D. Lond. two years later. He also passed the primary examination for the F.R.C.S. England.

After qualification he filled all the usual resident posts at the General Hospital, and having acquired a taste for the science of gynæcology, he took the post of House Surgeon at that well-known nursery of gynæcologists, the Jessop Hospital for Women, Sheffield, under another Bristolian, his friend Dr. Miles H. Phillips.

Returning to Bristol, he settled in practice at St. Andrew's Park, and joined the staff of the Bristol Dispensary, and also became Medical Officer to the Lying-in Hospital, Kingsdown. He afterwards resigned from the Dispensary on being appointed Visiting Medical Officer to Müller's Orphanage, Ashley Down, a post he still held when his illness laid him aside.

He also became Curator of the Museum and Surgical Registrar at the General Hospital. The latter post carried with it the duties of Registrar in the Gynæcological Department, in which his special knowledge stood him in good stead, and there is little doubt that he would in due course have taken his place on the honorary staff in that department. His knowledge of the subject was extensive, and based, as such knowledge must always be, on a sound knowledge of pathology.

In 1913 he removed to Clifton, and laid the foundation of what would have in time become an extensive special practice.

Early in 1914, however, there became manifest the first symptoms of the illness which prevented his taking any share in the military medical work which fell to his contemporaries. He was treated at several sanatoria, and although at one time an improvement in his health allowed him to accept a post as assistant medical officer at a sanatorium in Kent, the abatement was only a temporary one, and he returned home, only as it proved to die, early this year.

Pinniger was of a retiring disposition, and took no part in public affairs. He was, however, keenly interested in politics, and was a man of deep religious convictions. He was always an optimist, and although "spes phthisica" has become proverbial, it was difficult for the writer, in visiting him in more than one sanatorium and seeing the well-known lanky form and smiling face, to realise that he was, as he knew, in the grip of a mortal disease.

Dr. Pinniger married a twin daughter of the late Rev. W. Blake Atkinson, of Weston-super-Mare, and to his widow and three young children the deepest sympathy of the local profession will go out in their grievous loss.

